# Immunohistochemical expression of PD-L1 and its correlation with microsatellite status in endometrial and ovarian clear cell carcinomas: a cross-sectional study

**DOI:** 10.1186/s12885-022-10478-7

**Published:** 2022-12-29

**Authors:** Dorsa Ghasemi, Fereshteh Ameli, Fatemeh Nili, Ramtin Edjtemaei, Shahrzad Sheikhhasani

**Affiliations:** 1 Department of Pathology, Imam Khomeini Hospital Complex, Tehran University of Medical Sciences, End of Keshavarz Ave, Tehran, IR Iran; 2 Department of Gynecology Oncology, Imam Khomeini Hospital Complex, Tehran University of Medical Sciences, Tehran, IR Iran

**Keywords:** Ovarian clear cell carcinoma, Endometrial clear cell carcinoma, PD-L1, Mismatch repair protein status

## Abstract

**Background:**

Clear cell carcinoma is an uncommon histologic subtype of ovarian and endometrial carcinoma with poor response to Platinium-based chemotherapy agents at high stages. Blockage of Programmed cell Death Ligand-1 (PD-L1), can be used in targeted immunotherapy. This study investigated Mismatch Repair Deficiency (MMR-D) status, PD-L1 expression, and the correlation between PD-L1 expression and microsatellite instability (MSI) status in ovarian and endometrial clear cell carcinomas.

**Methods:**

Ovarian clear cell carcinoma (OCCC) (*n* = 28) and endometrial clear cell carcinoma (ECCC) (*n* = 28) samples were evaluated for PD-L1 (in tumoral and peri-tumoral inflammatory cells), MSH6 and PMS2 expression by immunohistochemistry (IHC) study. PD-L1 expression > 1% in tumor cells and > 5% in peritumoral inflammatory cells were considered positive.

**Results:**

The prevalence of PD-L1 expression was higher in ECCC (20/28, 71.43%) compared to OCCC tumor cells (16/28, 57.15%) (p > 0.05), while expression in peritumoral inflammatory cells was significantly higher in ECCC (25/28, 89.29%) compared to OCCC (11/28, 39.28%) (*p* < 0.05). MMR-D was observed in 5 cases, four OCCCs and one ECCC, among which, four (80%) showed PD-L1 expression in peritumoral inflammatory and tumor cells. The only OCCC case with extensive PD-L1 expression in tumor cells (> 50%) exhibited MSH6/MSH2 loss. No significant correlation was noted between PD-L1 expression and the pathologic stage or survival.

**Conclusion:**

PD-L1 expression was significantly associated with clear cell morphology, especially in the endometrium, independent of MMR protein status.

## Introduction

Endometrial carcinoma is the most common, and carcinoma of the ovaries is the leading cause of death among gynecologic cancers [1]. Clear cell carcinoma comprises 10% of ovarian and 6% of endometrial cancers [1]. The common characteristic of ovarian clear cell carcinoma (OCCC) and endometrial clear cell carcinomas (ECCC) on microscopic examination is mixed architectural patterns (solid, tubulocystic, and papillary structures), stromal hyalinization, and clear or eosinophilic cytoplasm accompanied with variable nuclear atypia [2]. These histologic subtypes are associated with poor prognosis in advanced stages.

OCCC demonstrates a poor response to chemotherapy with platinum-based regimens; therefore, its prognosis is similar to undifferentiated carcinoma [3]. The prognosis of ECCC is even poorer compared to endometrioid carcinoma. ECCC often demonstrates aggressive clinical behavior and is associated with poor outcome [4]. These carcinomas are often accompanied by extrapelvic spread at diagnosis [5]. The main prognostic factors for ECCC include age, FIGO stage, tumor size, myometrial infiltration, lymphovascular invasion, distant metastasis, Ki-67 index, and P53 expression [6].

OCCC is mainly associated with stage 1, which has a similar prognosis with other carcinoma subtypes [7]. On the other hand, ECCC is usually associated with higher stages. As both the ECCC and OCCC are resistant to Platinum-based chemotherapy in advanced stages, their treatment is challenging [8]. Therefore, targeted therapy or immunotherapy is considered beneficial in these cancers [9].

Knowing the pathogenesis pathway and molecular changes in the carcinogenesis of OCCC and ECCC can help in identifying the predictors of drug resistance and treatment in these patients. The most common and the most important molecular changes in clear cell carcinoma are *KRAS*, *PTEN, PIK3,* and *AIRID1* mutations. In contrast to serous carcinoma, *BRACA* and *P53* mutations are not common [10].

Programmed cell death ligand (PD-L1) is a trans-membrane protein that is the main ligand in programmed cell death and a potent mechanism for potentially immunologic tumors to escape from the host immune system [11]. PD1 plays a role in immune system response regulation and maintaining immune self-tolerance that are involved in the prevention of autoimmunity and controlling T-cell reaction [12]. PD-L1 is necessary in normal immune responses; however, in the case of malignancy, PD-L1 may provoke the disease. PD-L1 expression and its correlation with clinicopathologic features have been investigated in gynecologic cancers [13, 14]. But the data regarding the role of PD-L1 in CCCs are limited.

This study investigated the prevalence of MMR-D, PDL1 expression, and the correlation between PD-L1 expression with clinicopathologic features and MSI status in OCCC and ECCC.

## Materials and methods

Data from 80 cases with the diagnosis of OCCC and ECCC were collected through an electronic search in the hospital information system (HIS) of the Pathology Department of the Cancer Institute of Imam Khomeini Hospital Complex (IKHC), Tehran, Iran from 2016 to 2019. The study was approved by the local ethics committee of the university (IR.TUMS.IKHC.REC.1399.445). All cases were reviewed by two gynecologic pathologists using a multi-headed microscope to ascertain the initial diagnosis and to select the proper paraffin block. The main diagnostic criteria for OCCC and ECCC diagnoses were the identification of a mixture of papillary, tubulocystic, or solid patterns, thick hyaline basement membrane, and hobnail or clear cell features. An immunohistochemistry study for Napsin-A was performed in cases of equivalent morphologic features. Cases with other diagnoses (10 cases), inappropriate paraffin blocks (4 cases), and cases that received neo-adjuvant treatment (2 patients) were excluded. Finally, 28 OCCC cases and 28 ECCC cases were enrolled.

**Fig. 1:**
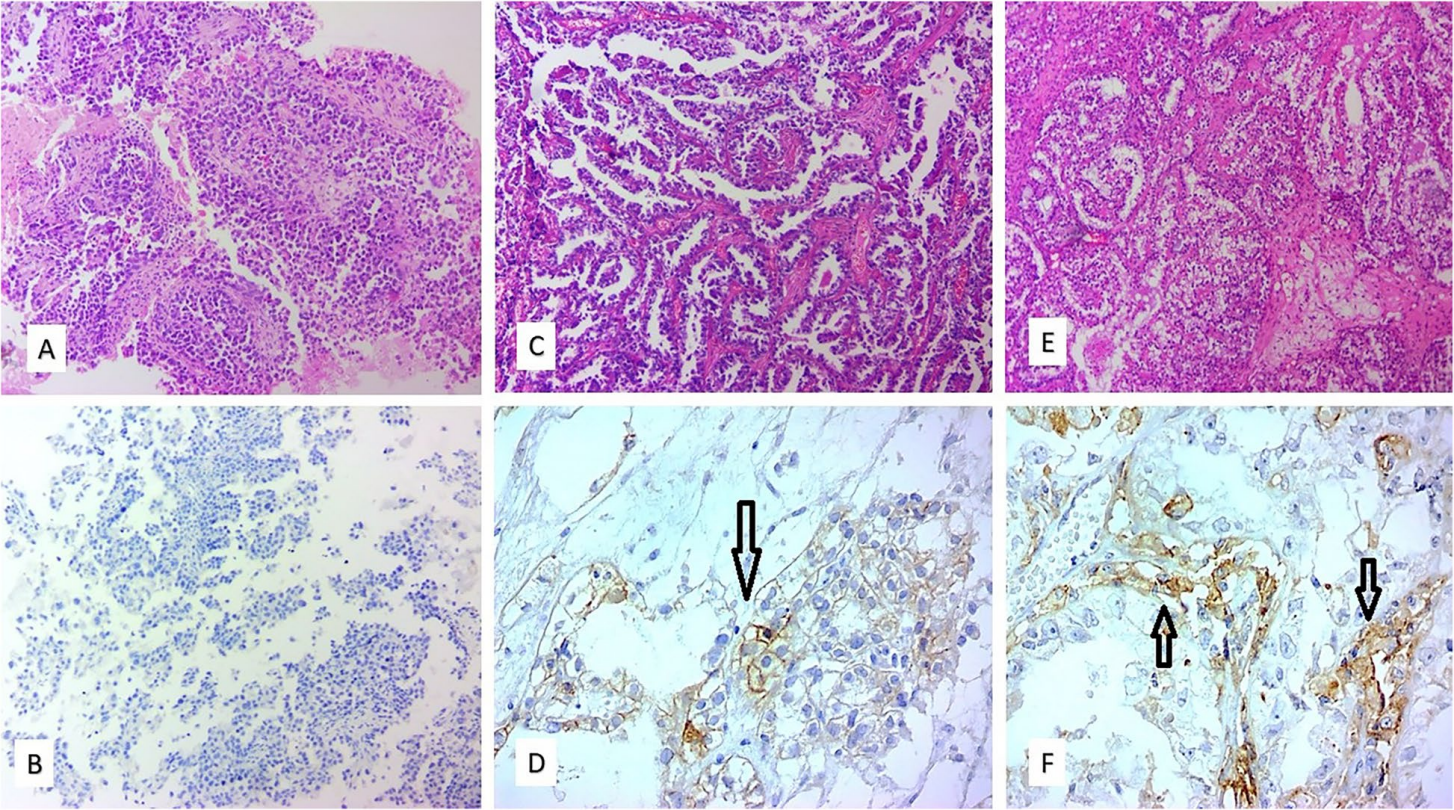
Microscopic examination of Hematoxylin and Eosin and IHC stained sections show Ovarian and Endometrial CCC with variable degree of PD-L1 expression in tumor cells (arrows): **A**, **B** Negative for PD-L1, **C**, **D** 1–5%, **E**, **F**  6–10%

**Fig. 2:**
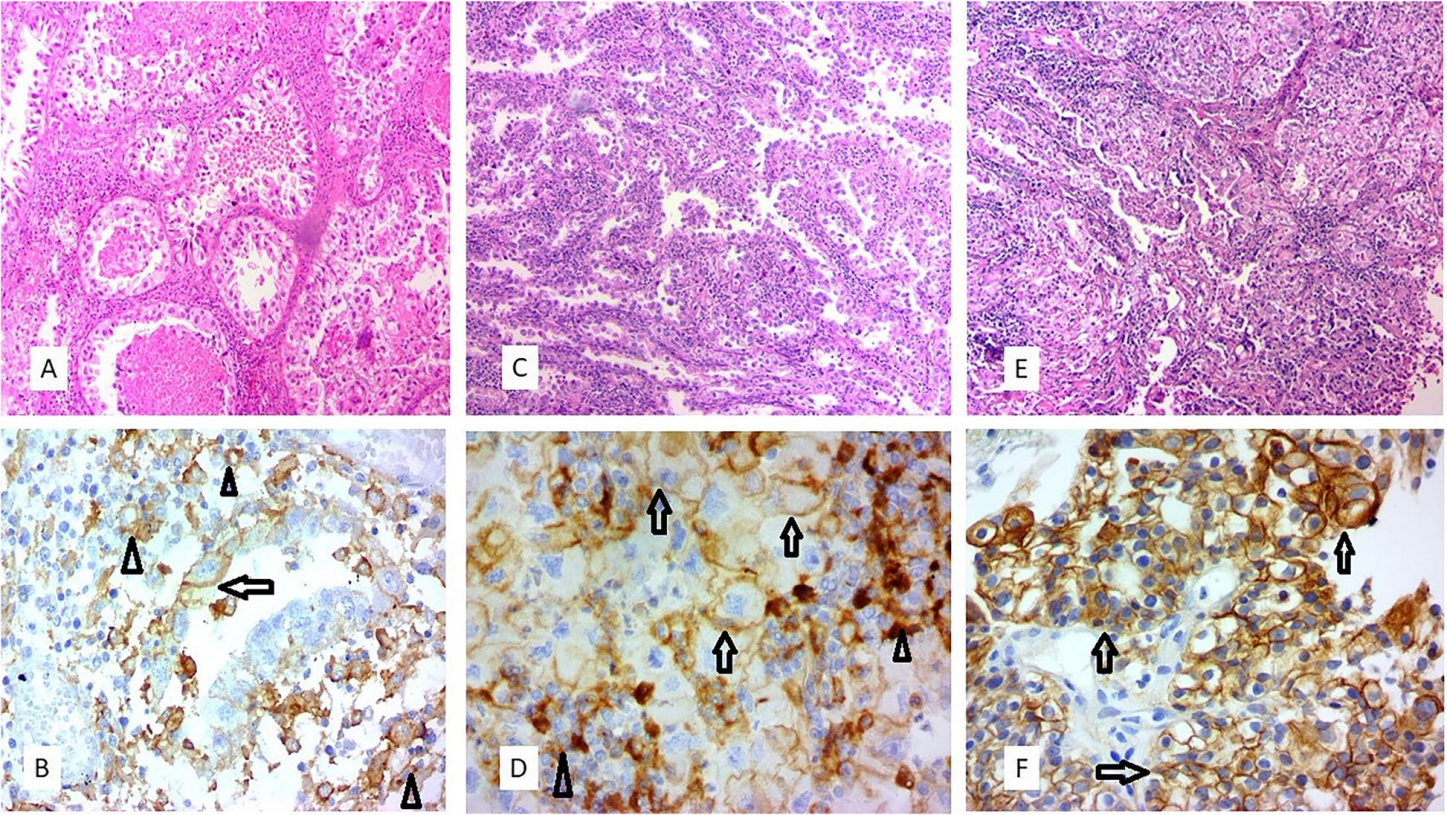
Microscopic examination of Hematoxylin and Eosin and IHC stained sections show Ovarian and Endometrial CCC with variable degrees of PD-L1 expression in tumor cells (arrows) **A**, **B** 11–24%, **C**, **D** 25–50%, **E**, **F** > 50%, extensive. In figures **B**, **D** peritumoral inflammatory cells with positive PD-L1 expression are also shown (arrow heads)

After preparing 3-$\mu m$-thick unstained slides, immunohistochemical staining for the PD-L1 marker by polymeric biotin-free horseradish peroxidase method was performed. The method had already been validated in our lab using tonsil tissue in which strong membranous positivity for this marker was observed in crypt epithelium as well as weak to moderate membranous positivity in follicular macrophages. After deparaffinization and rehydration, the antigen retrieval step was achieved by using Tris–EDTA buffer with PH = 8 at 100°c. Then the primary antibody PD-L1 (Rabbit Anti-Human Monoclonal Antibody, clone SBC-992, Sina Biotech) was added followed by the second antibody, and the final step was done using chromogen. The PD-L1 staining was performed on the whole sections of paraffin blocks. The PD-L1 staining was scored in both tumor cell and stromal inflammatory cells (peritumoral inflammatory) components. PD-L1 staining was considered positive if more than or equal to 1% of tumoral cells showed circumferential membranous staining. Positive PD-L1 staining was categorized based on the extent of staining into 1–5%, 6–10%, 11–25%, 25–50%, and > 50% (extensive staining) based on a previous study [13] (Fig. 1, 2). The expression of PD-L1 in intraluminal content and necrotic areas were disregarded. PD-L1 expression was patchy in tumor cells and intra-tumoral heterogeneity was notable in tumor cell components. The peritumoral inflammatory component reactivity was considered positive if more than 5% of immune inflammatory cells, including lymphocytes and macrophages, showed membranous or cytoplasmic staining with PD-L1. Positive PD-L1 cases were categorized into 5–10%, 10–50%, and > 50% (Fig. 1, 2).

All of the cases were assessed for their mismatch repair (MMR) status using the tissue microarray technique by extracting 4 mm cylindrical tissue samples from the paraffin blocks. The blocks were re-embedded into a new recipient block, then the initial IHC workup for PMS2 and MSH6 was performed. In cases with loss of each marker, further evaluation for partner proteins, “MLH1 and MSH6”, was performed. MMR deficient (MMR-D) status was defined as complete loss of nuclear staining for any of the four MMR proteins despite the presence of intact internal control (stromal lymphocyte staining). The postoperative patient survival, including overall survival (OS) and disease-free survival (DFS), was investigated 3 to 6 years after surgery.

Statistical analysis was carried out using the Chi-square test, Kaplan–Meier, and Cox regression analysis tests. Data were analyzed using the statistical package for statistical sciences (SPSS) version 26. *P* value < 0.05 was considered statistically significant.

## Results

The mean age of the patients in this study was 57.29 ± 12.58 years. There was no significant statistical difference in the mean age of the patients between OCCC (51.15 ± 9.63 years) and ECCC (63.44 ± 12.21 years). The pathologic stage was significantly higher in ECCC compared to OCCC (*P* = 0.043) (Table 1).

**Table 1:** Age, pathologic stage, and PD-L1 expression in OCCC and ECCC cases with MMR intact and MMR-D status

	**OCCC**			**ECCC**			
	MMR-D	MMR intact	total	MMR-D		MMR intact	total
**Age (mean ± SD)**	45.5 ± 7.8	52.18 ± 9.57	51.15 ± 9.63	35	64.62 ± 10.97		63.44 ± 12.21
**Pathologic stage(FIGO)**							
**I**	3	11	14	0	7		7
**II**	0	6	6	0	7		7
**III**	1	5	6	1	3		4
**IV**	0	0	0	0		0	0
**NA**	0	2	2	0		10	10
**PDL1 expression**							
**Tumor cells**	3	13	16	1		19	20
**Inflammatory cells**	3	8	11	1		24	25
**Total (either tumor or inflammatory cells)**	4	14	18	1		24	25

### MMR Status

MMR-D was observed in four OCCC cases (14.3%) and one case of ECCC (3.6%). The mean age of the patients with MMR-D in OCCC was 45.5 ± 7.8 with no significant statistical difference with MMR-intact cases (52.18 ± 9.57 years) (*P* > 0.05). The only ECCC case with MMR-D was 35 years old which was younger compared to MMR intact cases (64.62 ± 10.97, *P* > 0.05). Among the 4 MMR-D patients with OCCC, 3 patients demonstrated MSH6/MSH2, and one case showed MLH1/PMS2 loss. The only ECCC case with MMR-D exhibited loss of MSH2/MSH6. Three out of four patients with OCCC and MMR-D status were in FIGO stage I, while the other patient was in stage III. The pathologic FIGO stage of the only patient with MMR-D ECCC was III (Table 1).

### PD-L1 expression in OCCC and ECCC

Among the OCCC group, 18 cases (62.3%) showed either tumor cell or peritumoral inflammatory PD-L1 reactivity. Of those, 7 cases (38.9%) had only tumor cell reactivity, 2 cases (11.1%) had only peritumoral inflammatory reactivity and 9 cases (50%) were positive in both tumor and peritumoral inflammatory cells (Tables 2,3). In one case, extensive tumor cell staining (> 50%) with PD-L1 was noted.

**Table 2:** Frequency of PD-L1 expression in Ovarian and Endometrial clear cell carcinoma, tumor cells

**OCCC: Tumor cell staining with PD-L1**	Number and percentage of cases	**ECCC: Tumor cell staining with PD-L1**	Number and percentage of cases
**Negative (< 1%)**	42.85% (12/28)	**Negative (< 1%)**	28.57% (8/28)
**1–5%**	28.57% (8/28)	**1–5%**	39.28% (11/28)
**6–10%**	10.71% (3/28)	**6–10%**	10.71% (3/28)
**11–25%**	7.15% (2/28)	**11–25%**	14.3% (4/28)
**26–50%**	7.15% (2/28)	**26–50%**	3.57% (1/28)
** > 50%**	3.57% (1/28)	** > 50%**	3.57% (1/28)

**Table 3:** Frequency of PD-L1 expression in Ovarian and Endometrial clear cell carcinoma (OCCC) (ECCC) inflammatory cells

**OCCC: Peritumoral inflammatory staining**	Number and percentage of cases	**ECCC: Peritumoral inflammatory staining**	Number and percentage of cases
**Negative (< 5%)**	60.72% (17/28)	**Negative (< 5%)**	10.71% (3/28)
**5–10%**	25% (7/28)	**5–10%**	32.15% (9/28)
**10–50%**	10.71% (3/28)	**10–50%**	39.28% (11/28)

**Fig. 3:**
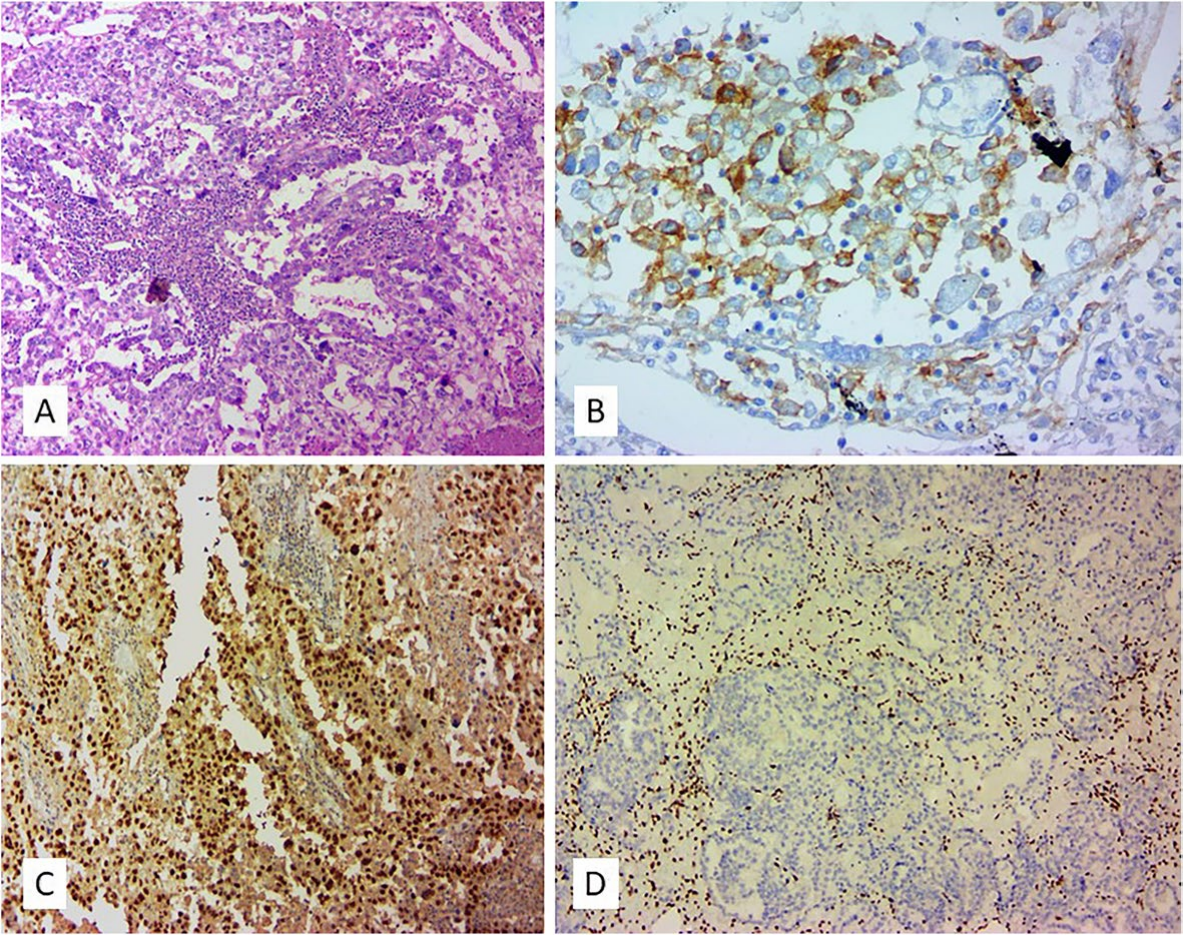
**A** A case of OCCC with MMR-D status, **B** PD-L1 expression in 11–25% of tumor cells, **C** MSH6 loss, D) PMS2 loss;

Among the ECCC group, 25 cases (89.3%) showed either tumor cell or peritumoral inflammatory PD-L1 reactivity. Of these, 20 cases (80%) were positive in both tumor and peritumoral inflammatory cells and 5 cases (20%) had only peritumoral inflammatory cell reactivity. Only three cases did not show peritumoral inflammatory reactivity with PD-L1 (Table 2, 3). The only ECCC case with MMR-D showed extensive staining for PD-L1 (50%) (Fig. 3). The ECCC groups also showed heterogeneous and patchy PD-L1 reactivity in tumor cells. In 5 cases with extensive (> 50%) peritumoral inflammatory PD-L1 expression, one showed MMR-D.

### Comparison of PD-L1 expression between OCCC and ECCC

ECCC showed more cases with PD-L1 expression in tumor cells (20/28) compared to OCCC (16/28) but the difference was not statistically significant (*P* > 0.05). PD-L1 expression in peritumoral inflammatory cells was significantly higher in ECCC (25/28) compared to OCCC (11/28) (*P* < 0.001).

### MMR status and PD-L1 expression

Three out of 4 cases of OCCC with MMR-D showed PD-L1 expression in tumor cells, among which the only case of OCCC had extensive PD-L1 expression (> 50%). Peritumoral inflammation was found in all four cases, among which 3 cases showed PD-L1 expression in the peritumoral inflammatory component. The only case of OCCC with > 50% PD-L1 expression in the peritumoral inflammatory component showed MMR-D (Table 4).

**Table 4:** MMR-D cases, their PD-L1 expression status and other pathologic characteristics

MMR-D status	Patient age (year-old)	Tumor PD-L1	Peritumoral inflammation	Peritumoral inflammatory PD-L1	Pathologic stage(FIGO)
OCCC, MSH2/6 loss	46	Negative (< 1%)	> 50%	5–10%	I
OCCC, MSH2/6 loss	44	> 50% (extensive)	50%	10–50%	III
OCCC, MSH2/6 loss	35	11–25%	5%	Negative (< 5%)	I
OCCC, MLH1/PMS2 loss	57	1–5%	40%	> 50%	I
ECCC, MSH2/6 loss	35	11–25%	> 50%	> 50%	III

The only case of ECCC with MMR-D demonstrated PD-L1 expression in both tumor cell and peritumoral inflammatory cell components. PD-L1 reactivity was 11–25% in tumor cells and > 50% in peritumoral inflammatory cells (Table 4).

No significant relationship was observed between PD-L1 expression in tumor cells and MMR status (in OCCC and/or ECCC) and between PDL-L1 expression in peritumoral inflammatory cells and MMR status (in OCCC and/or ECCC) (*P* > 0.05).

### PD-L1 expression and clinicopathologic features

No significant relationship was observed between lymphovascular invasion as well as pathologic stage and PD-L1 expression in either OCCC or ECCC cases (*P* > 0.05). Clinical data were available for 48 cases, including 23 OCCC and 25 ECCC. Forty percent (40%) of the cases died (8 ovary and 11 endometrial CCC patients). The median overall survival of OCCC and ECCC was 39 (95% CI: 21.4–56.5) and 45 (95% CI: 28.8–61.1) months, respectively (*p* > 0.05). No significant statistical relationship was found between PD-L1 expression in either tumor cells or peritumoral inflammatory cells and patient mortality (*P* > 0.05). The log-rank between PD-L1 positive and negative OCCC and ECCC in tumoral and peritumoral inflammatory cells was > 0.05 (Table 5).

**Table 5:** Median OS and DFS in OCCC and ECCC and its relationship with PD-L1 expression

Tumor site	PD-L1 (tumor cells or inflammatory cells)	Median overall survival (95% CI)	Median disease-free survival (95% CI)
Ovary	Positive	46 (22.3–69.6)	46 (22.6–69.6)
	Negative	31 (0–65.2)	31
Endometrium	Positive	45 (39.8–50.1)	31 (0–77.3)
	Negative	33 (0–85.8)	3 (0–7.8)

## Discussion

In our study, 4 OCCC cases (14.3%) and one ECCC case (3.6%) showed MMR-D. Studies about the prevalence of microsatellite instability in OCCC and ECCC are limited. In a study by Lorenzi et al., the prevalence of MMR-D and MSI-H was assessed in various solid tumors. The study reported that 25% of endometrial carcinomas and 11% of ovarian carcinomas were MMR-D. The endometrioid and clear cell subtypes were mostly associated with the microsatellite instability [15]. Some studies have emphasized the importance of routine screening for MMR status in patients with OCCC. Vierkoetter et al., concluded that patients younger than 53 years with clear cell or endometrioid ovarian carcinomas were at clinically significant risk for MMR-D; thus routine screening was recommended [16]. The study reported MMR-D in 7 out of 90 patients (7.7%) with both OCCC and ECCC. MMR-D was observed in 20% of patients under 53 years of age [16]. The frequency of MSI-H/MMR-D in OCCC was reported 21% and 5.5% by Cai et al. and Bennet et al., respectively [17]. In the study of Zhang et al. 17% of ECCC were MMR-D [6]. In a recent study by Cao and his colleagues, only one out of 20 ECCC cases (5%) was MMR-D [18]. According to the new World Health Organization (WHO) classification of female genital tract tumors, 0–6% of OCCCs and 0–33% ECCCs are MMR deficient [19]. Significant variation in different previous studies may be due to demographic differences, technical issues in IHC or molecular studies, and variations in the interpretation of results, as well as accurate pathologic diagnosis of CCC. The diagnosis of clear cell subtype especially in the endometrium is challenging. Endometrioid carcinoma with secretory features or serous carcinoma with clear cell changes may be misinterpreted as clear cell carcinoma [1]. In this study, two expert gynecopathologists reviewed the cases and the IHC marker, Napsin-A, was applied to confirm the diagnoses in equivocal cases. Although a higher number of our OCCC cases were MMR deficient, due to the small number of cases, we couldn’t analyze the significance of this difference with ECCC cases.

In our study, the most common pattern of MMR-D was MSH2/MSH6 loss; 4 out of 5 patients with MMR-D had MSH6/2 loss and only one of the OCCC cases showed MLH1/PMS2 deficiency. A similar pattern was observed in previous studies. MSH2/6 deficiency was found in all of three MMR-D OCCC cases in the study by Willis et al. Among 4 of the 21 MMR-D ECCC cases, two had MSH2/MSH6, one case had MSH6 and only one case had MLH1/PMS2 loss [13]. All of the 5/28 MMR-D ECCC in the study by Zhang et al. revealed loss of MSH2 which was associated with loss of MSH6 in one of the cases [6]. This pattern of MMR protein deficiency shows a significant correlation between MMR-D OCCC and ECCC with Lynch syndrome rather than somatic mutations [20].

PD-L1 expression in different ovarian and endometrial tumors and its relationship with prognosis have been widely studied. Studies regarding PD-L1 expression in clear cell carcinoma subtype are limited. We especially selected this histologic subtype, due to its therapeutic challenge with conventional chemotherapy drugs. In our study, 18 OCCC (64.28%) and 25 ECCC cases (89.28%) demonstrated PD-L1 expression in either tumor or peritumoral inflammatory cell components. Tumor cell reactivity with PD-L1 was observed in 16 OCCC and 20 ECCC cases; however, this difference was not statically significant (*P* > 0.05). In a similar study in 2017, PD-L1 reactivity in tumor cells was significantly higher in ECCC (76%, 16/21) compared to OCCC (43%, 10/23) [13]. Besides PD-L1 expression in tumor cells, we also evaluated PD-L1 reactivity in peritumoral inflammatory cells. Twenty-six cases of OCCC (92.85%) and 27 cases of ECCC (96.42%) demonstrated peritumoral inflammation. Among these cases, PD-L1 expression in the peritumoral inflammatory component was significantly higher in ECCC (25, 89.28%) compared to OCCC (11, 39.28%) (*P* < 0.001). In the aforementioned similar study, peritumoral inflammatory reactivity with PD-L1 was seen in 52% of OCCC cases and 76% of ECCC cases; however, this difference was not statistically significant [13]. Although molecular changes and protein expression are very similar in OCCC and ECCC, PD-L1 expression can be different due to distinct peritumoral environment in the endometrium causing more immune system stimulation and higher PD-L1 expression in either tumor cell or peritumoral inflammatory cells, compared to the ovary. Ultimately, due to higher stages of ECCCs and more prevalence of PD-L1 expression in these tumors, anti-PD-L1 inhibitor drugs may be more advisable. This hypothesis should be validated in future clinical trial studies.

The correlation between PD-L1 expression and MMR status in CCCs was the other goal of our study to investigate. MMR protein deficiency leads to microsatellite instability, increased mutation load in cancer-related genes, and formation of neoantigens that stimulate higher host immune response against the tumor [21]. PD-L1 is expressed due to high inflammatory cell infiltrate. Anti-PD-L1 drugs can improve survival, especially in MMR-D cancers [13]. In our study, three out of four OCCCs (75%) and the only ECCC case with MMR-D (100%), showed PD-L1 expression in tumor cells and peritumoral inflammatory cells. Three out of a total of five ECCC and OCCC cases with MMR-D had more than or equal to 50% peri-tumoral inflammation and one OCCC case showed extensive PD-L1 expression in tumor cells. In a similar study on 23 OCCC cases, MMR-D was reported in three cases. In two cases, PD-L1 was positive in tumor cells and these two cases were the only cases that had 10–25% PD-L1 staining. None of the cases showed peritumoral inflammatory reactivity for PD-L1. From 21 ECCC cases, 4 showed MMR-D, among which 3 cases had tumor cell PD-L1 expression, and 3 cases showed PD-L1 expression in peritumoral inflammatory cells. One of the ECCC cases with MMR-D showed extensive PD-L1 expression in tumor cells [13]. Howitt et al. studied 30 patients with OCCC in 2017. All the 3 MMR-D cases in the study showed some degree of PD-L1 expression in tumor or peritumoral inflammatory cells. Among the microsatellite stable cases, 44.4% expressed PD-L1 in tumor cells/ peritumoral inflammatory cells [22]. In the study of Matsuura et al. 108 out of 125 (86%) of all OCCCs were positive for PD-L1 [23]. In the study by Willis, the majority of MMR-intact ECCC cases showed PD-L1 expression. Alldredge et al. realized that clear cell phenotype, including uterine and OCCC express PD-L1 and have high PD-1 expression within tumor lymphocytes, which may correlate with tumor stage [24]. These findings were in line with the findings of our study. Despite a higher expression of PD-L1 in MMR-D CCCs, a significant number of MMR-intact tumors (64.2% of OCCC and 89.2% of ECCCs in our study) were positive for PD-L1 either in tumor cells or peritumoral inflammatory cells. Some researchers believe that clear cell morphology can be considered as a biomarker for PD-1/PD-L1 inhibitors regardless of MMR status [13]. Although the only one case with extensive PD-L1 expression, showed deficiency of MMR proteins, it is difficult to conclude the relationship between MMR-D and extensive PD L1 expression, due to limited number of the cases.

Although higher PD-L1 expression in tumor and inflammatory cells can be predictive of a good response to immunotherapy against PD-1/PD-L1 inhibitor drugs, this relationship was not confirmed in all studies. The reason for this different clinical response may be due to the differences in antibodies against these markers and differences in IHC staining methods. Therefore, there is a need for establishing common protocols and laboratory accreditation for the confirmation of IHC results with molecular tests or investigating the prognostic impacts and therapeutic responses comparing the IHC and mRNA expression of PD-L1 [25]. Moreover, the relationship between PD-L1 expression in a tumor and response to immunotherapy should be more precisely evaluated. In a recent clinical trial study, immunotherapy against PD-L1 improved survival in ECCC tumors that expressed PD-1/PD-L1, especially in MMR-deficient cases. In this study, from 20 ECCC cases, 6 showed PD-L1 expression in stromal lymphocytes, and in 3 showed PD-L1 reactivity in tumor cells. One case was MMR deficient. This study demonstrated that more PD-L1 expression was associated with higher stages and increased myometrial invasions [18]. The effect of the anti-PD-L1 antibody (Nivolumab) was studied in Platinum resistant ovarian carcinomas. Among 20 cases with complete response to anti-PD-L1 treatment, two cases were OCCC [26]. Other studies have shown benefits for immunotherapy against PD1/PD-L1 in advanced carcinomas, especially in MMR-deficient cases. In a study, the effectiveness of an anti-PD-L1 inhibitor (Pembrolizumab) in the treatment of metastatic solid tumors and Nivolumab for the treatment of colorectal cancers with MMR-D were confirmed [21].

Although most studies recommend the threshold of 1% for tumor cell PD-L1 positivity and initiation of immunotherapy, this threshold should be more exclusively evaluated.

Finally, we investigated the prognostic impact of PD-L1 expression in ovarian and endometrial CCCs. No significant statistical correlation between the expression of PD-L1 and tumor stage, site, lymphovascular invasion, OS, and DFS was found. Wang et al. reviewed 12 studies including 1630 cases of ovarian carcinoma. The meta-analysis didn’t show the association of PD-L1 expression by IHC study with tumor subtype, stage, grade, lymph node metastasis, and overall or disease-free survivals [27]. While no association between PD-L1 mRNA expression and OS was found, mRNA expression was significantly correlated with worse progression-free survival (PFS) [27]. In a study in 2021, decreased DFS was observed in endometrial carcinoma cases with immune cell PD-L1 positivity ≥ 5% [28]. In another study, the relationship between PD-L1 expression and prognosis was studied among 120 OCCC patients. PD-L1 expression was accompanied by an advanced pathologic stage, positive ascitic fluid, and increased recurrence. OS was also associated with a lower PD-L1 expression [29]. Matsuura et al. reported worse PFS and OS in OCCCs with positive PD-L1 expression. In the study of Alldredge and colleagues, no IHC expression pattern of PD-L1 was predictive of OS or DFS in multivariate analysis in OCCC and ECCC. In this study, stage III carcinomas revealed higher expression of PD-L1 than the stage I/II and IV cases [24]. The controversial results of previous studies suggest a need for further clinical investigations to validate the prognostic impact of PD-L1 in OCCC and ECCCs.

## Conclusion

Clear cell carcinoma morphology independent of MMR status is associated with PD-L1 expression in tumor cells and/or peritumoral stromal inflammatory cells in ovarian and endometrial CCC. This expression was higher in endometrial carcinoma, which is more commonly diagnosed in advanced stages.

## Data Availability

The data that support the findings of this study are available from the Hospital information system of Imam Khomeini Hospital affiliated with TUMS but restrictions apply to the availability of these data, which were used under license for the current study and so are not publicly available. Data are however available from the corresponding author (F. N) on reasonable request with permission of the TUMS.

## Abbreviations

OCCC: Ovarian clear cell carcinoma
ECCC: Endometrial clear cell carcinoma
PD-L1: Programmed Death Ligand-1
PD-1: Programmed Death-1
IHC: Immunohistochemistry
FIGO: International Federation of Gynecology and Obstetrics
MMR proteins: Mismatch repair proteins
MSI: Microsatellite instability
MMR-D: Mismatch repair deficient
NA: Not applicable
OS: Overall survival
DFS: Disease-free survival
PFS: Progression-free survival
